# Entwicklung einer Onlineintervention zum Thema Suizidalität – Vermittlung von Wissen und Reduzierung von Suizidstigma

**DOI:** 10.1007/s00103-021-03471-1

**Published:** 2021-12-13

**Authors:** Mareike Dreier, Julia Ludwig, Johanna Baumgardt, Martin Härter, Olaf von dem Knesebeck, Thomas Bock, Sarah Liebherz

**Affiliations:** 1 Institut und Poliklinik für Medizinische Psychologie, Zentrum für Psychosoziale Medizin, Universitätsklinikum Hamburg-Eppendorf, Martinistr. 52, 20246 Hamburg, Deutschland; 2 Institut für Medizinische Soziologie, Zentrum für Psychosoziale Medizin, Universitätsklinikum Hamburg-Eppendorf, Hamburg, Deutschland; 3 Klinik und Poliklinik für Psychiatrie und Psychotherapie, Zentrum für Psychosoziale Medizin, Universitätsklinikum Hamburg-Eppendorf, Hamburg, Deutschland; 4 Klinik für Psychiatrie, Psychotherapie und Psychosomatik, Vivantes Klinikum Am Urban, Berlin, Deutschland

**Keywords:** Suizidprävention, Suizidstigma, E‑Mental-Health, Online-Intervention, Trialog, Betroffenenbeteiligung, Suicide prevention, Suicide stigma, E‑mental-health, Online intervention, Lived experience, Peer involvement

## Abstract

Bei Suizidalität können die Angst, von anderen stigmatisiert zu werden, sowie Selbststigmatisierung und unzureichende Informationen dazu führen, dass Hilfsangebote weniger oder gar nicht in Anspruch genommen werden. E‑Mental-Health-Interventionen sind eine Möglichkeit, niederschwellig viele Betroffene über die Thematik zu informieren und auf persönliche Hilfsangebote vorzubereiten. Am Universitätsklinikum Hamburg-Eppendorf wurde eine komplexe Intervention entwickelt, gefördert im Rahmen des Förderschwerpunkts „Suizidprävention (A: Entstigmatisierung)“ des Bundesministeriums für Gesundheit. Entwicklung und Inhalte dieser Onlineintervention sollen im vorliegenden Beitrag beschrieben werden.

Nach einer repräsentativen Telefonbefragung der deutschen Allgemeinbevölkerung, mit der Wissenslücken und Stigmatisierungstendenzen zum Thema Suizid untersucht wurden, entstand auf Basis eines australischen Suizidpräventionsprojekts und in Zusammenarbeit mit Betroffenen und Angehörigen die Onlineintervention „8 Leben – Erfahrungsberichte und Wissenswertes zum Thema Suizid“. Darin wurden sowohl wissenschaftlich-klinische Fakten rund um das Thema Suizidalität als auch eine gesellschaftlich-kulturelle Perspektive beleuchtet sowie auf Selbsthilfemöglichkeiten und professionelle Hilfsangebote verwiesen. Es wurden Videoerfahrungsberichte von Betroffenen und Angehörigen gezeigt. Aktuell wird das Projekt ausgewertet. Eine Weiterführung ist geplant.

In der Intervention werden verschiedene Sichtweisen gezeigt und die Personen, die die Intervention in Anspruch nehmen, werden sowohl auf kognitiver als auch auf emotionaler Ebene angesprochen. Aufgrund der Prävalenz von Suizidalität und der dennoch bestehenden Tabuisierung des Themas scheinen seriöse, evidenzbasierte und niedrigschwellige Präventions- und Informationsangebote besonders relevant.

## Abstract (en)

Fear of being stigmatized by others, self-stigmatization, and inadequate information can limit those affected by suicidality from seeking help. E‑mental-health interventions provide a low-threshold way to reach many individuals with information about the topic. This enables those affected to prepare themselves for personal offers of help. As part of the funding priority “Suicide Prevention (A: Destigmatization)” of the German Federal Ministry of Health, a complex intervention was developed at the University Medical Center Hamburg-Eppendorf. The development and content of this online intervention are described in this article.

Following a representative telephone survey of the German general population, which investigated gaps in knowledge and stigmatization tendencies on the subject of suicide, the online intervention “8 lives – lived experience reports and facts on suicide” was developed on the basis of an Australian suicide prevention project and involved persons with a lived experience of suicide. The intervention highlighted both scientific and clinical facts about suicidality as well as a socio-cultural perspective and offered self-help options and professional support services. Video reports of persons with a lived experience of suicide were shown within the intervention. The project is currently being evaluated. A continuation is planned.

The intervention is a multi-layered offer in which different perspectives on the topic of suicidality are presented. Participants are addressed on a cognitive and emotional level. Given the prevalence of suicidality and the fact that the subject is still taboo, serious, evidence-based, and low-threshold prevention and information offers seem particularly relevant.

## Hintergrund

Jedes Jahr sterben weltweit rund 800.000 Menschen durch Suizid [1, 2]. Im Jahr 2016 entsprach dies einer altersstandardisierten Rate von 10,5 auf 100.000 Einwohner:innen. Europa war mit einer Rate von 12,9 die Region mit der zweithöchsten Rate weltweit, nach Südostasien mit 13,4. In Deutschland lag die altersstandardisierte Rate im selben Jahr bei 9,1, wobei ein erheblicher Unterschied zwischen Männern (13,6) und Frauen (4,8) bestand [2]. Ungefähr 25 Menschen nehmen sich jeden Tag in Deutschland das Leben [3]. Die Zahl der Suizidversuche wird etwa 10- bis 20-mal so hoch eingeschätzt [1].

Die Mehrheit der Suizide in den westlichen Industrieländern erfolgt vor dem Hintergrund einer psychischen Störung [4, 5], vor allem einer Depression. Bei ca. der Hälfte aller vollendeten Suizide liegt in der Krankheitsgeschichte eine affektive Störung vor [4, 6]. Obgleich evidenzbasierte Behandlungsmöglichkeiten für psychische Störungen und suizidale Krisen zur Verfügung stehen [7], führen Angst vor Stigmatisierung, Selbststigmatisierung und unzureichende Informationen dazu, dass betroffene Personen weniger Hilfsmöglichkeiten in Anspruch nehmen, Suizidalität geheim halten und sich selbst bzgl. ihrer Suizidalität abwerten [8–12]. So erhalten von den ca. 16 Mio. erwachsenen Bundesbürger:innen, die in einem Jahr an einer psychischen Störung leiden, nur 36 % eine diesbezügliche Behandlung. Bei nur 10 % von ihnen kann dabei von einer adäquaten Behandlung ausgegangen werden [13].

Deshalb sollten für Menschen, die keinen Zugang zur Versorgung finden können oder wollen, niedrigschwellige, barrierefreie Angebote zur Verfügung gestellt werden, um sich über die eigene Problematik zu informieren und die Gesundheitskompetenz zu verbessern. Das US National Institute of Mental Health [14] hat zum Beispiel die Entwicklung innovativer Behandlungsansätze empfohlen, die sowohl weniger Kosten verursachen als auch einer großen Population zugänglich sind.

Mit der mittlerweile großen Verbreitung moderner Kommunikationstechnologien (2019/2020 sind 86 % der Bevölkerung Deutschlands Internetnutzer:innen [15]) ergeben sich neue Möglichkeiten, die Versorgung von Menschen in psychischen Krisen zu verbessern. So zeigt eine Übersichtsarbeit, dass die Nutzung neuer Medien (Computer, Smartphones, Tablets) auch bei psychischen Störungen wirksam im Hinblick auf eine Verbesserung des Therapieerfolgs sein kann [16]. Internetbasierte Anwendungen können helfen, sich über psychische Störungen zu informieren, das Ausmaß der eigenen Betroffenheit abzuschätzen, lokale Behandlungsangebote zu finden und sich auf den Kontakt mit Behandler:innen vorzubereiten. Selbsthilfeprogramme können darüber hinaus signifikant zur Besserung der Symptomatik beitragen. Beispielsweise existieren für Betroffene mit depressiven Störungen mittlerweile einige Selbsthilfeprogramme [17].

Bezüglich Suizidalität können Aufklärungskampagnen helfen, Stigmatisierung zu reduzieren und die Offenheit gegenüber Hilfsangeboten zu fördern [18]. Eine aktuelle Übersichtsarbeit zu Suizidpräventionsstrategien fand zudem Evidenz für Zugangsrestriktionen (beispielsweise Absperrungen an hohen Gebäuden oder Brücken), für Aufklärungskampagnen in Schulen, für spezifische psychopharmakologische und psychotherapeutische Verfahren sowie für die Nachsorge von Betroffenen mit Suizidversuchen [7]. Zu E‑Mental-Health-Ansätzen liegen bislang zu wenige Studien vor, um deren Wirksamkeit zu beurteilen.

Das Ziel dieses Artikels ist es, sowohl die Entwicklung als auch die Inhalte der Onlineintervention „8 Leben – Erfahrungsberichte und Wissenswertes zum Thema Suizid“ detailliert zu beschreiben. Die Intervention entstand im Rahmen des Projekts *Entwicklung und Evaluation von E‑Mental-Health-Interventionen zur Entstigmatisierung von Suizidalität (4E) *am Universitätsklinikum Hamburg-Eppendorf. Das Projekt wurde im Rahmen des Förderschwerpunkts „Suizidprävention (A: Entstigmatisierung)“ des Bundesministeriums für Gesundheit von Oktober 2017 bis Dezember 2019 gefördert. Speziell ist, dass es sich dabei nicht um eine therapeutische Intervention im klassischen Sinne handelt. Vielmehr wurde gemeinsam mit Betroffenen eine Intervention entwickelt, die durch Aufklärung und Sensibilisierung zur Suizidprävention beitragen soll. Im Folgenden wird an entsprechender Stelle auf bereits publizierte Daten, die im Rahmen des Projekts entstanden sind, verwiesen.

## Entwicklung der Onlineintervention

Zur Entwicklung der Onlineintervention wurde zunächst eine telefonische Bevölkerungsbefragung durchgeführt. Als Vorlage für die Onlineintervention diente das australische Suizidpräventionsprojekt *The Ripple Effect*. Die Inhalte wurden evidenzbasiert gestaltet und in Zusammenarbeit mit Betroffenen und Angehörigen erstellt. Am Ende erfolgte die technische Umsetzung. Nun konnten Personen die Intervention nutzen. In die Intervention waren Fragebögen zur Evaluation integriert, zudem folgte danach mit einigen Teilnehmenden ein Interview zur Evaluation. Im Folgenden werden die einzelnen Elemente der Entwicklung näher beschrieben.

### Telefonische Bevölkerungsbefragung

Als Grundlage für die Entwicklung der Onlineintervention wurde im April und Mai 2018 zunächst eine computergestützte telefonische Befragung (CATI) in der erwachsenen Bevölkerung (18 Jahre und älter) in Deutschland durchgeführt, um suizidspezifisches Wissen und Einstellungen gegenüber Menschen mit Suizidgedanken in der Gesellschaft zu erfassen. Dabei kamen standardisierte und validierte Befragungsinstrumente aus der Stigmaforschung zum Einsatz sowie Instrumente zur Erfassung suizidspezifischen Stigmas und Wissens. Die Befragung wurde vom Sozialforschungsinstitut USUMA (Berlin) durchgeführt. Für Interviews in Haushalten mit mehreren Zielpersonen wurde eine Zufallsauswahl der Zielperson über den Schwedenschlüssel (Kish-Selection-Grid) realisiert. Bei dieser Methode wurde die Auswahl der Befragungsperson nach Aufnahme aller im Haushalt lebenden Zielpersonen programmtechnisch und unabhängig von Interviewerentscheidungen über ein Zufallsverfahren realisiert. Zu Beginn des Interviews wurden die Teilnehmenden über Nutzen der Studie aufgeklärt sowie über die ausschließlich zweckgebundene und anonyme Verwendung ihrer Daten (informierte Einwilligung, engl.: „informed consent“; [19–21]).

**Figure 1:**
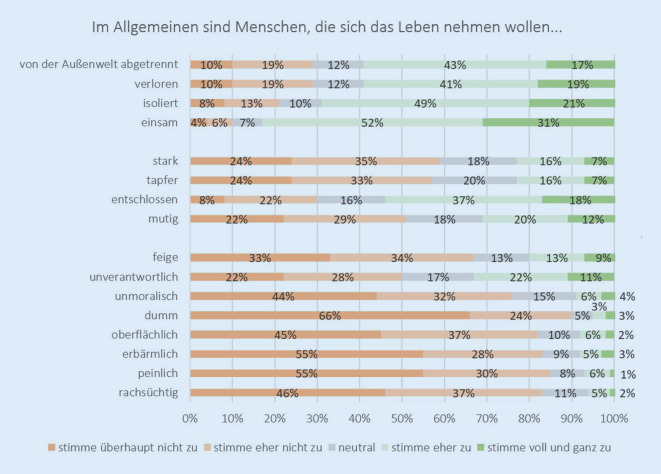

Insgesamt haben *N* = 2002 Personen an der Befragung teilgenommen, was einer Antwortquote von 47,3 % entspricht. Ein Vergleich mit der offiziellen Statistik zeigte, dass die Verteilung der demografischen Charakteristika Geschlecht, Alter oder Bildung nicht signifikant von der Verteilung in der Allgemeinbevölkerung abweicht [22]. Die Befragten wurden unter anderem gebeten, vermeintliche Eigenschaften von Menschen (Stereotypen), die sich das Leben nehmen wollen, zu bewerten. Geringe Zustimmung zeigten die Befragten dabei gegenüber Eigenschaften, wie z. B. „erbärmlich“, „peinlich“ oder „dumm“. Höhere Zustimmung äußerten die Befragten bei Eigenschaften wie „isoliert“, „verloren“ oder „einsam“ (Abb. 1).

Weiterhin konnten mit der Auswertung verschiedene Themenbereiche identifiziert werden, die später in der Onlineintervention adressiert wurden, beispielsweise spezifische Wissenslücken (z. B. ältere Männer als besonders gefährdete Gruppe) und weitere stigmatisierende Einstellungen in der Gesellschaft (z. B. Menschen mit Suizidgedanken als „willensschwach“ wahrzunehmen). Weitere Ergebnisse der Befragung wurden andernorts publiziert [19–21].

### Zusammenarbeit mit dem australischen Projektteam von *The Ripple Effect*

Die Onlineintervention wurde auf Basis der bereits bestehenden australischen Onlineintervention* The Ripple Effect, *welche zur Suizidprävention bei männlichen Landwirten, entwickelt wurde [24, 25]. Es entstand eine interaktive, sprachliche, kulturelle und inhaltliche Adaption. Die Zielgruppe wurde verbreitert, da unsere Onlineintervention möglichst viele Bevölkerungsgruppen ansprechen sollte. Eine Beratung bei der Entwicklung erfolgte durch die Projektleitung von *The Ripple Effect *im Rahmen eines mehrtägigen Workshops sowie mehrerer Videokonferenzen und E‑Mails.

### Trialogische Entwicklung

Betroffene (Personen mit Suizidgedanken oder Suizidversuchen) sowie Angehörige (Personen, die eine nahestehende Person durch Suizid verloren haben, und Personen, die sich um eine nahestehende suizidale Person sorgen) sollten in die Entwicklung der Onlineintervention einbezogen werden. Zusammen mit den Expert:innen fand ein Erfahrungsaustausch in einer trialogischen Arbeitsgruppe statt, die über den Verein „Irre menschlich Hamburg e. V.“ gegründet wurde [26], der von Psychiatrieerfahrenen, Angehörigen und Therapeut:innen getragen wird. *Irre menschlich Hamburg* organisiert Informations‑, Begegnungs- und Präventionsprojekte zu allen Aspekten von seelischer Gesundheit, psychischer Erkrankung und Anderssein und wirbt für mehr Toleranz im Umgang mit anderen und mehr Sensibilität für sich selbst.

Nachdem das Projektvorhaben in einer Vereinssitzung vorgestellt wurde, konnten sich interessierte Betroffene und Angehörige für eine Mitarbeit entscheiden (sofern sie mindestens 18 Jahre alt waren). Diese Personen erhielten eine schriftliche und mündliche Projektbeschreibung und Studieninformation, ihr psychischer Zustand wurde von psychologischen Psychotherapeut:innen und ihnen selbst als ausreichend stabil für eine Teilnahme eingeschätzt und sie unterschrieben eine Einwilligungserklärung. Jede Person hatte zu jeder Zeit das Recht, ihre Beteiligung inkl. Bereitstellung ihres Video- und Textmaterials zu widerrufen.

Die trialogische Arbeitsgruppe bestand aus insgesamt 10 Personen (*n*_Frauen_ = 6, *n*_Männer_ = 3, *n*_divers_ = 1) im Alter von 19–73 Jahren, die über verschiedene Erfahrungen mit Suizidalität bzw. Suizid verfügten (*n*_Betroffene_ = 7, *n*_Angehörige_ = 3), unterschiedlichen Arbeitsstatus (*n*_Studierende_ = 1, *n*_Angestellte_ = 4, *n*_Erwerbslose_ = 1, *n*_Vorruhestand_ = 2, *n*_Rentner:innen_ = 2) sowie unterschiedliche Berufe hatten (z. B. Lehrerin, Arzt, Lkw-Fahrer, Sekretärin, Disponent, ohne Berufsausbildung, Sozialwissenschaftlerin). Bis zur Fertigstellung der Onlineintervention fanden insgesamt 12 Arbeitsgruppentreffen statt, die von den Wissenschaftler:innen und Kliniker:innen des Projekts moderiert und koordiniert wurden. Die Texte, die für die Intervention erstellt wurden (Verweis auf externe Hilfsangebote, Wissen über Suizid und Suizidalität, Suizidversuche verstehen, Stigmatisierung und Tabu, Irrtümer über Suizid, Strategien zum Umgang mit Suizidalität, Ziele setzen), durchliefen eine strukturierte schriftliche und mündliche Prüfung (Review) mit beteiligten Betroffenen, Angehörigen und allen beteiligten Expert:innen des Projektteams.

**Figure 2:**
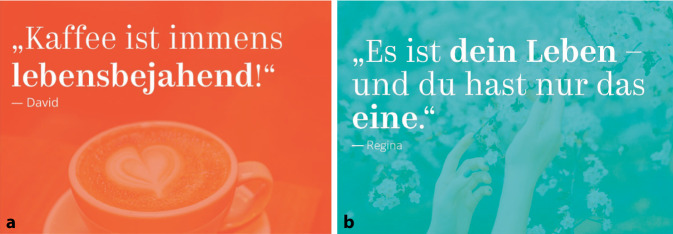

Die Arbeitsgruppenmitglieder konnten sich also beteiligen:

- an der Entwicklung von Konzept, Struktur, Inhalt und Gestaltung der Onlineintervention,
- an dem strukturierten Reviewprozess der Textmaterialien der Intervention,
- als Protagonist:innen an der Erstellung von persönlichen Video- oder schriftlichen Erfahrungsberichten und
- an der Erstellung von kurzen „digitalen Postkartenbotschaften“, die später innerhalb der Onlineintervention dargestellt werden sollten (Abb. 2).

6 Betroffene und 2 Angehörige (Tab. 1) berichteten in Videos über ihre Erfahrung mit Suizidalität. Die Erfahrungsberichte der 8 Personen waren später namensgebend für die Onlineintervention. Eine weitere Beteiligte der Arbeitsgruppe, die sich um eine nahestehende suizidale Person sorgte, schrieb einen Erfahrungsbericht zu Suizidalität aus ihrer Perspektive. Ein Arbeitsgruppenmitglied entschied sich gegen einen Erfahrungsbericht in Video- oder Textform. Alle 10 Arbeitsgruppenmitglieder beteiligten sich an der Erstellung der „digitalen Postkartenbotschaften“. Als laienverständlicher Name für die Onlineintervention entschieden sich die Arbeitsgruppe und die Projektmitarbeitenden für „*8 Leben – Erfahrungsberichte und Wissenswertes zum Thema Suizid*“ und nutzten den Begriff „Onlineprogramm“ statt „Onlineintervention“ [27].

**Table 1:** 

	Erfahrungshintergrund der Protagonist:innen in den Videos	Altersgruppe, Geschlecht Protagonist:in
1	Einmaliger Suizidversuch nach einem Verkehrsunfall	70–80 Jahre, männlich
2	Einmaliger Suizidversuch im frühen Erwachsenenalter nach einer Trennung	60–70 Jahre, weiblich
3	Suizidgedanken in einer depressiven Episode	20–30 Jahre, männlich
4	Wiederkehrende Suizidgedanken	40–50 Jahre, weiblich
5	Wiederkehrende Suizidgedanken in Krisen	50–60 Jahre, weiblich
6	Chronische Suizidalität mit Suizidversuchen	40–50 Jahre, divers
7	Verlust der Mutter durch Suizid	50–60 Jahre, männlich
8	Verlust des Vaters durch Suizid	30–40 Jahre, weiblich

### Evidenzbasierung

Bei der Entwicklung der Onlineintervention wurden das Manual „Gute Praxis Gesundheitsinformation“ [28] sowie weitere internationale und nationale Qualitätskriterien zur Erstellung von evidenzbasierten Gesundheitsinformationen und Entscheidungshilfen^1^ berücksichtigt (siehe dazu auch Methodenpapier psychenet [29]). Auf Basis dieser Standards wurden die Interventionsmaterialien erstellt. Als Quellen für die Informationstexte der Intervention wurden die S3-Leitlinie „Nationale Versorgungsleitlinie Unipolare Depression“ [30] und die NICE-Leitlinie „Self-harm in over 8 s: long-term management“ [31] herangezogen. Zusätzlich wurden bei der Erstellung die Leitlinien zur Berichterstattung über Suizid [32] berücksichtigt. Zudem erfolgte eine eigene Recherche nach aktuellen systematischen Übersichtsarbeiten in Literatur- und Zitierdatenbanken. Es wurde eine enge Betroffenenbeteiligung realisiert (siehe Abschnitt „trialogische Entwicklung“) sowie Transparenz, Aktualisierung und Interessenneutralität beachtet.

### Technische Umsetzung der Onlineintervention

**Figure 3:**
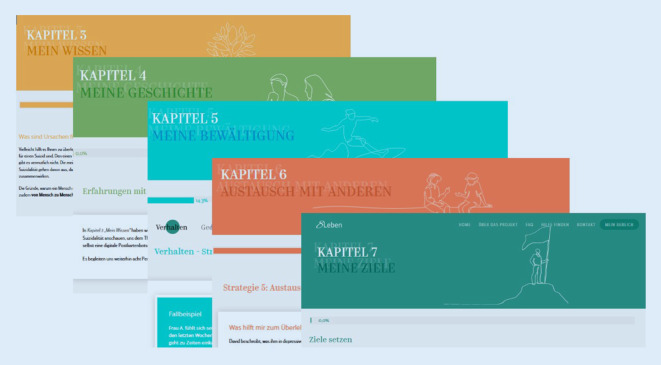

Die technische Umsetzung sowie die Gestaltung (Abb. 3) erfolgten durch eine Webdesignagentur. Die Betroffenen- und Angehörigengruppe, die Wissenschaftler:innen und Kliniker:innen sowie Mitarbeiter:innen des Instituts und der Poliklinik für Medizinische Psychologie prüften die Onlineintervention vor Liveschaltung in 2 Testdurchläufen hinsichtlich des allgemeinen Nutzungserlebnisses sowie inhaltlicher und technischer Auffälligkeiten bzw. Fehler.

### Evaluation der Onlineintervention

Die interaktive Onlineintervention wurde in einem Mixed-Methods-Design mit 3 Messzeitpunkten (Prä, Post, Follow-up) evaluiert. Primäre bzw. sekundäre Outcomes wurden definiert: (a) Verbesserung des Wissens bzgl. Suizidalität, (b) Veränderungen der Selbststigmatisierung bzw. wahrgenommene Fremdstigmatisierung bzgl. Suizidalität und (c) Veränderung der Selbstwirksamkeitserwartung, mit psychisch belastenden Situationen umgehen zu können. Zielgruppe der Onlineintervention waren Erwachsene (≥ 18 Jahre), die als Betroffene oder Angehörige Erfahrungen mit dem Thema Suizidalität bzw. Suizid gemacht haben. Andere Interessierte konnten ebenfalls teilnehmen [33].

Die Teilnehmer:innen wurden über verschiedene Wege auf die Studie zur Onlineintervention aufmerksam gemacht. Dazu gehörten u. a. Teaser auf dem E‑Health-Portal psychenet.de (ca. 120.000 Besucher:innen pro Monat; Koordination, Pflege und Weiterentwicklung der Website vom Institut und Poliklinik für Medizinische Psychologie am Universitätsklinikum Hamburg-Eppendorf) [34], E‑Mails an verschiedene Multiplikatoren mit der Bitte, den Link zur Studienwebsite zu verteilen (z. B. an Kliniken, Stiftungen, Selbsthilfeorganisationen, Landespsychotherapeutenkammern), Hinweise auf die Studie in Onlineforen und sozialen Medien. Eine Suchmaschinenoptimierung (SEO) wurde durchgeführt, um die Website besser auffindbar zu machen. Zusätzlich zur Onlinerekrutierung wurden Poster, Postkarten und Aushänge in Supermärkten, Arztpraxen und psychiatrischen Einrichtungen in Hamburg und Berlin verteilt. Teilnehmende der Onlineintervention wurden schriftlich über die Studie und Verwendung ihrer Daten informiert und konnten im Anschluss ihr Einverständnis durch Ankreuzen eines Onlinekästchens geben. Die Auswertung und Publikation zur Evaluation sind aktuell in Vorbereitung.

Die Evaluationsergebnisse fließen in die Verstetigung der Onlineintervention mit ein, z. B. werden dazu Verbesserungsvorschläge der Teilnehmenden systematisch ausgewertet und berücksichtigt.

## Aufbau und Inhalte der interaktiven Onlineintervention

Zu Beginn der Intervention wurde die eigene Erfahrung mit dem Thema Suizidalität bzw. Suizid erfragt. Je nach Art der Betroffenheit wurden die Teilnehmenden einem von 5 Interventionspfaden zugeordnet (Tab. 2). Die Inhalte und die Auswahl der angezeigten Erfahrungsberichte variierten dementsprechend leicht.

**Table 2:** 

	Pfad	Art der Betroffenheit bzgl. Suizidalität und/oder Suizid
Betroffene	1	Selbst betroffen durch aktuelle Suizidgedanken oder Suizidgedanken in der Vergangenheit
	2	Selbst betroffen durch einen oder mehrere Suizidversuche
Angehörige	3	Als Angehörige:r betroffen durch den Verlust einer nahestehenden Person durch Suizid
	4	Als Angehörige:r betroffen durch die Sorge um eine nahestehende, suizidale Person
Interessierte	5	Allgemein an der Thematik Suizidalität interessiert, z. B. durch die Arbeit in einem Gesundheitsberuf

### Inhalte der interaktiven Onlineintervention

Tab. 3 zeigt eine Übersicht über die Inhalte der 8 Kapitel der interaktiven Onlineintervention, die bei der Nutzung sukzessive freigeschaltet wurden. Innerhalb der Kap. 1 und 2 und 8 wurden den Teilnehmenden Onlinefragebögen zur Evaluation der Intervention dargeboten.

**Table 3:** 

Kapitel	Beschreibung	Spezifizierung und Beispiele
Kapitel 01: ÜBER MICH	*In Kapitel 1 wurden ****soziodemografische Angaben**** erhoben und es erfolgte die ****Zuordnung**** der Teilnehmenden zu einem der 5 Pfade anhand der Angabe ****zur eigenen Erfahrung mit Suizidalität. ****Es wurde eine digitale Postkartenbotschaft je nach angegebener Erfahrung dargeboten*	Erfragt werden: Alter, Geschlecht, Bildungsgrad, Größe des Wohnorts, eigene Erfahrung mit Suizidalität bzw. Suizid, von der man am stärksten betroffen ist
		Beispieltext der **Postkartenbotschaft** Pfad 3 (Verlust durch Suizid): *„Ich habe eine mir sehr wichtige und geliebte Person durch Suizid verloren. Dieser Verlust hat mich sehr aus der Bahn geworfen und ich habe viel Zeit gebraucht, um damit klarzukommen. Mir hat es geholfen, mit mir nahen, vertrauten Menschen darüber zu sprechen und Zeit in der Natur zu verbringen.“ – Johanna*
Kapitel 02: MEINE GEDANKEN	*In Kapitel 2 wurden Messinstrumente zur ****Evaluation*** ***der Onlineintervention**** eingesetzt (****Prämessung****). Je nach Pfad wurden unterschiedliche Items angezeigt (Pfad 1 und 2: insgesamt 52 Items; Pfad 3, 4 und 5: insgesamt 35 Items)*	*Literacy of Suicide Scale* (LOSS-SF; [35]) zur Erfassung von **Wissen bzgl. Suizidalität**
		Adaption der *Stigma of Suicide Scale* (SOSS-SF; [21, 23]) zur Erfassung von **Selbststigmatisierung bzgl. Suizidalität** (Pfad 1 und 2) und **wahrgenommener Fremdstigmatisierung bzgl. Suizidalität** (alle Pfade)
		Adaption des* Distress-Thermometers *[36] zur Erfassung der aktuellen **Belastung**
		Selbst entwickelter Fragebogen orientiert am Selbstwirksamkeitskonzept von Bandura [37, 38] zur Erfassung der **Selbstwirksamkeitserwartung, mit belastenden Situationen umgehen zu können** (6 bzw. 7 Items, intervallskaliert), z. B.: „*Ich traue mir zu, über Verhaltensweisen zu sprechen, die mir langfristig nicht guttun (z.* *B. zu viel oder zu wenig schlafen oder essen, zu viel arbeiten, Alkohol‑/Drogenkonsum).“*
Kapitel 03: MEIN WISSEN	*In Kapitel 3 erfolgte eine ****Psychoedukation**** zu Suizidalität und Suizid sowohl anhand von Informationstexten als auch anhand von Videoerfahrungsberichten von Betroffenen und Angehörigen*	**Definition** Suizidalität
		**Kontinuumsannahme** von Suizidalität [39], dargestellt als Grafik
		Unterschiede in Häufigkeit und Intensität von Suizidalität anhand eines Beispiels mit einem Videoausschnitt *„Im Video berichtet Jenny, wann Suizidalität bei ihr stärker wird.“*
		**Ursachen** für Suizidalität anhand eines multifaktoriellen Erklärungsmodells [1], wobei ein Schwerpunkt auf belastende Lebensereignisse, gesellschaftliche und psychosozialen Faktoren gelegt wird; Videoausschnitte z. B.: *„Andre wollte sich nach einem schweren LKW-Unfall das Leben nehmen. Er berichtet im Video, warum er sein Leben beenden wollte“; „David berichtet im Video, warum Menschen aus seiner Sicht Suizidgedanken haben. Er berichtet auch von Phasen, in denen er selbst Schwierigkeiten hatte, Alltägliches zu erledigen.“*
		**Risiko- und Schutzfaktoren für Suizidalität **[40]
		Optional: Eigene Risiko- und Schutzfaktoren reflektieren (nur Pfad 1 und 2)
		**Warnsignale** für (akute) Suizidalität aus Sicht von Angehörigen und Betroffenen
		Exkurs Prävalenzen
		Exkurs zum Werther- und Papageno-Effekt [41]
		Exkurs zum Verzicht auf die Begriffe „Selbstmord“ und „Freitod“
Kapitel 04: MEINE GESCHICHTE	*In Kapitel 4 stellten sich 8 Betroffene und Angehörige mit Foto und Text vor. Stigmatisierung und Tabuisierung in Zusammenhang mit Suizidalität wurden erklärt und über Irrtümer aufgeklärt. Teilnehmende konnten ihre eigene Erfahrung mit Suizidalität in Form einer „digitalen Postkartenbotschaft“ mitteilen*	**8 Videoerfahrungsberichte sowie ein Textbericht** von Betroffenen und Angehörigen; Beispiele: „*Im Video berichtet Birgit über ihre Erfahrung mit dem Wunsch oder Drang, sich umbringen zu wollen, und wie sie Kraft geschöpft hat, weiterzuleben“*; „*Regina berichtet, wie sie mit ihrem Suizidversuch umgegangen ist, wie sie bei der Entscheidung fürs Leben bleiben kann und was sie anderen, die mit Suizidgedanken leben, mitgeben möchte.“*
		Definition **Stigma bzw. Stigmatisierung** und **Tabuisierung, **Gründe für und Auswirkungen von Stigmatisierung und Tabuisierung von Suizidalität [42–46]
		Aufklärung zu bekannten **Irrtümern** über Suizidalität [47], z. B. *Irrtum: „Über Suizid zu sprechen, bringt Menschen erst auf die Idee, sich das Leben zu nehmen.“ Wirklichkeit: „Fragen nach Suizidgedanken machen nicht suizidal: Entweder sind Gedanken an Suizid da oder nicht. Offen Suizidgedanken zu thematisieren, ist häufig eine Entlastung für alle Beteiligten. Zudem kann sie Betroffenen Möglichkeiten aufzeigen, wie sie mit ihrer Situation umgehen können. Wegen des Stigmas um Suizid wissen Betroffene oft nicht, mit wem sie sprechen können.“*
		Möglichkeit, **eigene Erfahrung mit Suizidalität bzw. Suizid** anonym **mitzuteilen** per „digitaler Postkartenbotschaft“ (Fragen: *„Welche Erfahrungen haben Sie mit dem Thema Suizidalität?“; „Was hilft Ihnen, mit dem Thema Suizid/Suizidalität besser umzugehen?“; „Was wünschen Sie sich von anderen Menschen im Umgang mit dem Thema Suizid/Suizidalität?“*)
		Exkurs: **Tatsächliche Stigmatisierung vs. Wahrgenommene Stigmatisierung**
		Exkurs: **Suizidalität bei Gruppen mit einem höheren Risiko für Stigmatisierung** (Migrationshintergrund, Intelligenzminderung, körperliche Erkrankung, höheres Alter, sexuelle Identität/Geschlechtsidentität)
Kapitel 05: MEINE BEWÄLTIGUNG	*In Kapitel 5 wurden kognitiv-behaviorale Selbsthilfestrategien zum Umgang mit eigener Suizidalität bzw. Suizidalität oder Suizid von Angehörigen vorgestellt. Das Konzept des Krisen- oder Notfallplans bei Suizidalität wurde eingeführt*	Erneuter Hinweis: Intervention ist keine Krisenintervention, Hinweis zu weiterführenden Hilfsangeboten in (suizidalen) Krisen
		Einführung des **Konzepts Notfallplan bei Suizidalität** und Verweis auf geprüfte Vorlagen von Krisen- und Notfallplänen (nur Pfad 1 und 2)
		Trauerrituale, Thema Schuld (jeweils nur Pfad 3)
		*Optional*: Strategien zum Umgang bzw. zur Vorbeugung von Suizidalität auf **Verhaltensebene** (Fallbeispiele zu Teufelskreisen, Aufbau positiver Aktivitäten), **Körperebene** (progressive Muskelrelaxationstechniken), **Kognitionsebene** (Hinterfragen von negativen Gedanken), und **Emotionsebene** (Gefühle wahrnehmen und benennen) inkl. jeweils eines PDF-Arbeitsblatts pro Bereich
		Ergänzende Videos aus Betroffenen- und Angehörigensicht (z. B.: „*Was hat mir geholfen, mit dem Suizid meines Vaters umzugehen?“; „Was kann in schwierigen Situationen helfen?“; „Was hilft mir mit Suizidalität umzugehen?“; Ausdrucksmöglichkeiten für Gefühle*)
Kapitel 06: AUSTAUSCH	*In Kapitel 6 wurden Strategien zur Kommunikation über Suizidalität bzw. den Verlust durch Suizid vorgestellt*	Strategien zur **Kommunikation** über Suizidalität mit unterschiedlichen Personengruppen wie Familie, Freund:innen, Ärzt:innen, Psychotherapeut:innen und Umgangsmöglichkeiten bei Schwierigkeiten und Formulierungsideen (z. B.: *„wie man in einem Arzt- oder Therapeutengespräch eigene Suizidgedanken ansprechen kann“;* [48])
		Videoerfahrungsberichte, z. B.: „*Johanna möchte anderen Menschen, die jemanden durch Suizid verloren haben, und Menschen, die suizidal sind, aus ihrer Erfahrung mitgeben, wie wichtig es ist, sich nach außen zu wenden*“; „*Im Video beschreibt David, was man machen kann, wenn man akute Suizidgedanken hat.“*
		Reflexion zur Offenlegung von Suizidalität und Suizidversuchen bzw. Suiziden von nahestehenden Menschen; Benennung von Vor- und Nachteilen einer Offenlegung
Kapitel 07: MEINE ZIELE	*In Kapitel 7 konnten Teilnehmende sich ein Ziel setzen, an das sie optional per E‑Mail erinnert wurden*	**Ziele** setzen nach *SMART*-Kriterien [49] bzgl. der vorgestellten Strategien in Kapitel 5 und 6. *SMART *ist ein Akronym für *S*pecific (spezifisch), *M*easurable (messbar), *A*ttractive (erstrebenswert), *R*ealistic (realistisch), *T*ime-bound (terminiert). Diese Beschreibungen können als Kriterien zur eindeutigen Definition von Zielen genutzt werden.
		Beispiele für Ziele: *„Gespräch mit Ärztin suchen: Beim nächsten Termin sage ich meiner Hausärztin, dass ich Gedanken habe, mir mein Leben zu nehmen, und dass ich mir Hilfe wünsche“; „Austausch mit anderen: Ich melde mich morgen per E‑Mail im Verein für Suizidtrauernde AGUS e.* *V., um mich dort für ein erstes Treffen anzumelden. Im Treffen kann ich über den Verlust meines Partners sprechen“; „Mehr Kontakt: Ich möchte wieder mindestens einmal in der Woche Freunde treffen. Dazu melde ich mich morgen Abend bei einer/m Freund/Freundin, verabrede mich für einen Abend zu zweit“; „Weniger Alkohol: Ich trinke montags bis donnerstags und sonntags keinen Alkohol mehr und versuche, freitags und samstags maximal 2 Bier zu trinken.“*
		Möglichkeit, eigenes Ziel zu setzen und daran per E‑Mail erinnert zu werden
Kapitel 08: FEEDBACK	*In Kapitel 8 wurden (wie in Kapitel 2) Messinstrumente zur ****Evaluation*** ***der Onlineintervention**** eingesetzt (****Postmessung****): *Wissen bzgl. Suizidalität, stigmatisierende Einstellungen gegenüber von Suizidalität Betroffenen, aktuelle Belastung und Selbstwirksamkeitserwartung, mit belastenden Situationen umgehen zu können. Zusätzlich wurde ein **Feedback **zur Intervention erhoben, hierbei wurde die Zufriedenheit mit der Intervention nach Abschluss quantitativ sowie qualitativ mittels Freitextantworten erhoben	*Literacy of Suicide Scale* (LOSS-SF; [35]) zur Erfassung von **Wissen bzgl. Suizidalität**, Adaption der *Stigma of Suicide Scale* (SOSS-SF; [21, 23]) zur Erfassung von **Selbststigmatisierung bzgl. Suizidalität** (Pfad 1 und 2) und **wahrgenommener Fremdstigmatisierung bzgl. Suizidalität** (alle Pfade), Adaption des* Distress-Thermometers *[36] zur Erfassung der aktuellen **Belastung, **selbst entwickelter Fragebogen orientiert am Selbstwirksamkeitskonzept von Bandura [37, 38] zur Erfassung der **Selbstwirksamkeitserwartung, mit belastenden Situationen umgehen zu können**
		Fragen zum **Feedback** umfassten Veränderung des **Wissens über Suizidalität und Veränderung von Stigmatisierungstendenzen** *(Bsp.: „Durch meine Teilnahme am Programm ‚8 Leben – Erfahrungsberichte und Wissenswertes zum Thema Suizid‘ kenne ich die Risikofaktoren und Schutzfaktoren von Suizidalität besser*.“); **Fertigkeiten*** (Bsp.: „Durch meine Teilnahme am Programm ‚8 Leben – Erfahrungsberichte und Wissenswertes zum Thema Suizid‘ kann ich besser mit anderen über Erfahrungen mit Suizid sprechen.“); ***hilfreiche Elemente der Intervention ***(„Bitte bewerten Sie, wie hilfreich Sie die einzelnen Elemente von ‚8 Leben‘ fanden: Videoerfahrungsberichte von Menschen, die Erfahrung mit Suizid haben“; „Was war am Programm ‚8 Leben‘ am hilfreichsten/am wenigsten hilfreich für Sie?“ mit optionalem Freitextfeld); ***Zufriedenheit mit der Länge des Programms; Auskunft zu Einstellungen bzw. Erfahrungen bzgl. Suizid/Suizidalität in der Intervention*** („Dies war das erste Mal, dass ich meine Einstellungen zum Thema Suizid und/oder Erfahrungen mit Suizid geteilt habe“; Antwortmöglichkeit: Ja/Nein; falls Nein: „Wo haben Sie bereits über Ihre Erfahrung gesprochen?“); ***Weiterempfehlung des Programms*** („Würden Sie das Programm ‚8 Leben‘ weiterempfehlen? Bitte begründen Sie warum.“)*

In den Kap. 3–7 der Onlineintervention waren jeweils mehrere kurze Videosequenzen von Interviewausschnitten der verschiedenen Betroffenen bzw. Angehörigen enthalten (Tab. 1**bzw.** 3). Diese berichteten über ihre Erfahrungen mit Suizidalität sowie darüber, was ihnen im Umgang mit Suizidalität bzw. dem Suizid einer nahestehenden Person geholfen hat. Die Länge der Interviewausschnitte variierte zwischen < 1 min und 12 min. Die Erfahrungsberichte fokussierten u. a. darauf, Hoffnung zum Weiterleben zu vermitteln, zu zeigen, wie man über das Thema Suizidalität und Suizid sprechen kann, und Unterstützungsmöglichkeiten in Krisen aufzuzeigen. Teilnehmende konnten während der Bearbeitung der Intervention Pausen einlegen. Es gab einen Navigationsbereich („Mein Bereich“), in dem man auf das zuletzt bearbeitete Kapitel und auf die verwendeten Quellen zugreifen konnte. Nachdem die Intervention abgeschlossen wurde, wurden die Videos mit den 8 Erfahrungsberichten in Gesamtlänge freigeschaltet und alle Arbeitsblätter zum Download zur Verfügung gestellt. Teilnehmende konnten hier außerdem auf die Postkartenbotschaften der anderen Teilnehmenden zugreifen. Die Postkartenbotschaften wurden vor Veröffentlichung geprüft, z. B. wurden Beschreibungen von Suizidmethoden aus den Botschaften entfernt. Die Onlineintervention war selbstangeleitet („unguided“), d. h., Teilnehmende erhielten keine persönliche Betreuung, wie z. B. Rückmeldungen per E‑Mail. Bei technischen Schwierigkeiten gab es die Möglichkeit, sich per E‑Mail mit dem Anliegen beim Studienteam zu melden. Während der gesamten Intervention war unter einem angezeigten Reiter „Hilfe finden“ ein Verweis zu verschiedenen persönlichen und telefonischen Hilfsangeboten eingeblendet.

## Diskussion

In der Bevölkerungsbefragung konnten Wissenslücken und Stigmatisierungstendenzen entdeckt werden. Diese wurden in der Onlineintervention adressiert.

Der breite Fokus der Onlineintervention auf verschiedene Zielgruppen ist eine Stärke der Intervention, stellte bei der Entwicklung aber auch eine Herausforderung dar, da verschiedene Perspektiven gleichermaßen berücksichtigt und sensibel adressiert werden sollten. Beispielsweise könnte es für Menschen, die nahestehende Personen durch Suizid verloren haben, weniger passend sein, Erfahrungsberichte von Betroffenen zu sehen, die eine suizidale Krise überwunden haben. In der trialogischen Arbeitsgruppe vertraten wir jedoch die Auffassung, dass das Verständnis der Gründe für eine suizidale Krise und einen suizidalen Zustand am glaubhaftesten von Betroffenen berichtet werden kann. Zudem wollten wir ein Verständnis für die Vielschichtigkeit von Suizidalität und Suizid fördern. Unser Projekt zeigte, dass der Einbezug von Betroffenen und Angehörigen in die Entwicklung einer Intervention auch bei komplexen und sensiblen Themen wie Suizidalität und Suizid sehr gut möglich und bereichernd ist. Die trialogische Arbeitsgruppe war an übergreifenden konzeptionellen Entscheidungen beteiligt, wie z. B. der Auswahl und dem Grad der Tiefe der Themen oder der Art und Weise, wie die Videoerfahrungsberichte den Teilnehmenden präsentiert werden, bis hin zu Detailentscheidungen wie der Formulierung von Sätzen [27]. Bislang wurden Personen mit Suiziderfahrung häufig nicht in Forschungsprojekte bzw. Interventionsentwicklungen miteinbezogen [50]. Die positiven Erfahrungen des vorgestellten Projekts können dazu ermutigen, diese Personen zukünftig stärker zu beteiligen.

In der vorgestellten Onlineintervention wurde der Komplexität des Themas Suizidalität und Suizid einerseits durch die trialogische Arbeitsweise begegnet und zudem wurde das Thema von verschiedenen Seiten beleuchtet: Es wurden wissenschaftliche Fakten rund um das Thema vermittelt, um sachlich aufzuklären. Andererseits adressierte die Intervention die Teilnehmenden auch auf emotionaler Ebene, beispielsweise durch die Erfahrungsberichte und die Möglichkeit, eigene Erfahrungen aufzuschreiben und mit anderen zu teilen. Des Weiteren wurden gesellschaftlich kulturelle Faktoren beleuchtet, beispielsweise die Entwicklung und Funktion der Tabuisierung des Themas sowie der „Werther-“^2^ und der „Papageno-Effekt“^3^ [41, 51].

Ein Schwerpunkt der Intervention lag überdies auf dem Thema Kommunikation rund um das Thema Suizidalität und Suizid, so sollten Teilnehmende einerseits ermutigt werden, sich Unterstützung zu suchen, gleichzeitig wurden sie angeleitet kritisch zu prüfen, wem in ihrem Umfeld sie sich unter welchen Bedingungen anvertrauen können. Selbsthilfestrategien, angelehnt an verhaltenstherapeutische Techniken, wurden angeboten. Dabei wurden die Ebenen Verhalten, Kognitionen, Körper und Emotionen einbezogen. Auch wurde den Teilnehmenden angeboten, ein Ziel zu einem der Bereiche oder zum Thema Kommunikation zu definieren, beispielsweise einen Termin für ein vertrautes Gespräch mit einer guten Freundin oder einem guten Freund zu vereinbaren und dort die aktuellen Sorgen anzusprechen.

Alle diese Angebote waren freiwillig, Teilnehmende konnten wählen, ob sie sich mit einem oder mehreren dieser Bereiche näher befassen wollten und ob sie ein Ziel definieren oder per E‑Mail daran erinnert werden wollten. Durch die fehlende persönliche Begleitung (es handelte sich bei der Onlineintervention um ein unbegleitetes Angebot, welches die Teilnehmenden selbstständig und ohne therapeutische Unterstützung durchliefen) könnte die Durchführung der angebotenen Selbsthilfestrategien für einige der Teilnehmenden eine Überforderung darstellen. Daher wurde im entsprechenden Kapitel darauf verwiesen, dass es derzeit überfordernd sein kann und es ggf. ratsam wäre, sich (professionelle) Unterstützung zu suchen. Gleichzeitig war während der gesamten Intervention ein Button mit Informationen zu professionellen Hilfsangeboten und Unterstützung in akuten Krisen verfügbar und es wurde darauf verwiesen, dass die Intervention keine Krisenintervention in einer akuten Gefahrensituation darstellt. Die wissenschaftliche Publikation zur Prä‑, Post- und Follow-up-Erhebung ist aktuell in Vorbereitung und wird im Laufe des Jahres erfolgen. Die Onlineintervention soll nach einer Aktualisierung, bei der auch die Rückmeldungen der Teilnehmenden berücksichtigt werden, auf der Subdomain https://8leben.psychenet.de/ des Portals *psychenet *[52] kostenlos zur Verfügung gestellt werden.

Durch diese unbegleitete Umsetzung konnten einerseits Teilnehmende in einer Krise nicht automatisch identifiziert und akut unterstützt werden. Gleichzeitig bot das frei verfügbare, kostenlose und jederzeit verfügbare Format ein besonders niederschwelliges Angebot und daher auch eine Möglichkeit für Menschen, die das persönliche Anvertrauen derzeit (noch) scheuten, sich mit dem Thema Suizidalität bzw. Suizid auseinanderzusetzen und Informationen über Unterstützungsmöglichkeiten zu bekommen.

## Fazit

Wissenslücken und Stigmatisierung bzgl. Suizidalität, die in der Bevölkerungsbefragung entdeckt wurden, wurden in der Onlineintervention adressiert. Die trialogische Entwicklung der Onlineintervention ist eine Besonderheit dieses Projekts. In authentischen Videoerfahrungsberichten verweisen Betroffene auf Hilfsmöglichkeiten, beschreiben den eigenen Umgang mit Suizidalität, zeigen persönliche Erfahrungen zur Überwindung von suizidalen Krisen auf und machten Mut zum Weiterleben. Die hier beschriebene Onlineintervention beleuchtet das Thema Suizidalität nicht nur von verschiedenen Standpunkten, sondern geht auch differenziert auf verschiedene Aspekte des Themas ein. So kommen sowohl wissenschaftliche psychologisch-psychotherapeutische und gesellschaftlich-kulturelle als auch philosophische Sichtweisen auf das Thema zum Tragen. Ähnliche Angebote fehlen in Deutschland aktuell vermutlich auch aufgrund der Tabuisierung von Suizidalität und der Sensibilität des Themas. In Anbetracht der Häufigkeit von Suiziden und Suizidversuchen, den schwerwiegenden Folgen für Betroffene und Angehörige auf der einen Seite und der Möglichkeit der Prävention von Suiziden und Behandelbarkeit von psychischen Störungen auf der anderen Seite erscheint uns eine Auseinandersetzung mit dem Thema Suizidalität im öffentlichen Raum in Form eines Angebots mit qualitätsgesicherten Informationen und authentischen Erfahrungsberichten zu Umgangsmöglichkeiten mit Suizidalität sehr relevant. Vor allem erscheint es wichtig, wissenschaftlich und auf hilfreiche und Hoffnung vermittelnde Art das Thema zu adressieren und somit unseriösen oder sogar gefährdenden Quellen im Internet (z. B. Suizidforen) etwas entgegenzusetzen.

## Footnotes

1 Z. B. www.discern.org.uk, https://www.healthonnet.org, http://ipdas.ohri.ca.

2 Werther-Effekt: Annahme, dass mediale Suiziddarstellungen zu einer Erhöhung der Suizidrate aufgrund von Nachahmungseffekten führt.

3 Papageno-Effekt: Annahme, dass eine angemessene mediale Berichterstattung über Suizide diese verhindern kann, z. B. durch den Verzicht auf monokausale Erklärungen und detailreiche Beschreibungen der Umstände und stattdessen Aufzeigen z. B. konstruktiver Bewältigungsmöglichkeiten von Krisensituationen und professioneller Unterstützungsmöglichkeiten.
